# Cotton Leafroll Dwarf Virus US Genomes Comprise Divergent Subpopulations and Harbor Extensive Variability

**DOI:** 10.3390/v13112230

**Published:** 2021-11-05

**Authors:** Roberto Ramos-Sobrinho, Raphael O. Adegbola, Kathy Lawrence, Drew W. Schrimsher, Thomas Isakeit, Olufemi J. Alabi, Judith K. Brown

**Affiliations:** 1 School of Plant Sciences, The University of Arizona, Tucson, AZ 85721, USA; ramosrs@email.arizona.edu (R.R.-S.); roadegbola@arizona.edu (R.O.A.); 2 Department of Entomology and Plant Pathology, Auburn University, Auburn, AL 36849, USA; lawrekk@auburn.edu (K.L.); drew.schrimsher@greenpointag.com (D.W.S.); 3 Department of Plant Pathology and Microbiology, Texas A&M University, College Station, TX 77843, USA; t-isakeit@tamu.edu; 4 Department of Plant Pathology and Microbiology, Texas A&M AgriLife Research and Extension Center, Weslaco, TX 78596, USA

**Keywords:** cotton viruses, genetic diversity, plant virus evolution, *Polerovirus*, *Solemoviridae*

## Abstract

Cotton leafroll dwarf virus (CLRDV) was first reported in the United States (US) in 2017 from cotton plants in Alabama (AL) and has become widespread in cotton-growing states of the southern US. To investigate the genomic variability among CLRDV isolates in the US, complete genomes of the virus were obtained from infected cotton plants displaying mild to severe symptoms from AL, Florida, and Texas. Eight CLRDV genomes were determined, ranging in size from 5865 to 5867 bp, and shared highest nucleotide identity with other CLRDV isolates in the US, at 95.9–98.7%. Open reading frame (ORF) 0, encoding the P0 silencing suppressor, was the most variable gene, sharing 88.5–99.6% and 81.2–89.3% amino acid similarity with CLRDV isolates reported in cotton growing states in the US and in Argentina and Brazil in South America, respectively. Based on Bayesian analysis, the complete CLRDV genomes from cotton in the US formed a monophyletic group comprising three relatively divergent sister clades, whereas CLRDV genotypes from South America clustered as closely related sister-groups, separate from US isolates, patterns reminiscent of phylogeographical structuring. The CLRDV isolates exhibited a complex pattern of recombination, with most breakpoints evident in ORFs 2 and 3, and ORF5. Despite extensive nucleotide diversity among all available CLRDV genomes, purifying selection (*dN/dS* < 1) was implicated as the primary selective force acting on viral protein evolution.

## 1. Introduction

Cotton (*Gossypium* spp.) is an economically important fiber crop grown in over 80 countries worldwide, mainly for use in the textile industry [1,2,3]. In 2019, global production of cotton exceeded 82 million tons, harvested from a >38-million-hectare cultivation area, with an estimated value of $74.4 billion dollars [4]. In the United States (US), 12 million tons of cotton were produced, representing a market value of $5.8 billion US dollars [5].

Bacteria, fungi, nematodes, and viruses are important limiting factors to cotton production globally [6]. The cotton blue disease (CBD) was first described in Central African Republic (CAR) in 1949, where cotton plants exhibiting virus-like symptoms were associated with infestations of the cotton aphid, which was implicated as the vector of the suspected viral pathogen [7,8]. Virus-like diseases were also described in aphid-infested cotton-growing fields in Brazil in 1938 and 1962 [9,10], and in the Misiones Province of Argentina during 1982–1983 [11]. Based on the similar foliar symptoms and transmission by the cotton aphid, the former diseases were hypothesized to share a similar undetermined virus-like etiology [12].

During 2003–2010, symptoms reminiscent of CBD in CAR were observed in commercially grown cotton in Brazil [12], East Timor [13,14], India [15], and Thailand [16]. Molecular characterization of partial and complete genome sequences from similarly symptomatic cotton plants revealed the presence of an aphid-transmitted polerovirus named cotton leafroll dwarf virus (CLRDV) [12,14,15,17,18]. Since the first report of CLRDV infecting cotton in Alabama, US, during 2016–2017 [19], CLRDV isolates have been reported from symptomatic cotton in Georgia, Mississippi, North Carolina, Texas, Arkansas, Florida, Louisiana, Oklahoma, and South Carolina [20,21,22,23,24,25,26,27,28,29,30]. Thus, CLRDV has become recognized as an emerging polerovirus [31,32]. Plants infected with CLRDV may develop shortened internodes, and thus the extent of overall stunting may range from extensive to none. Infected cotton leaves may develop reddening of leaf blades and petioles, curling and downward cupping, blue green, intense green, or red rust-coloration, vein-yellowing, or blistering, with yield loss attributed to reduced boll set [14,18,19,32,33]. Perhaps unexpectedly, CLRDV has been found to infect non-cotton hosts, including several uncultivated plant species [34].

*Cotton leafroll dwarf virus* belongs to the genus *Polerovirus* (family *Solemoviridae*). The virus has a linear, positive-sense, single-stranded, monopartite RNA genome of ~5.8 kb in size, encapsidated in a spherical virion of approximately 23 nm in diameter [35,36]. As for other poleroviruses, CLRDV is phloem limited. To date, the only insect vector identified is the cotton aphid (*Aphis gossypii* Glover), which transmits the virus in a persistent, circulative, non-propagative manner [8,37,38]. The CLRDV genome organization resembles that of other poleroviruses [12,17,32,39], consisting of seven open reading frames (ORFs) partitioned into two regions separated by a 200-nucleotide (nt) intergenic region (IR) that functions in the synthesis of sub-genomic RNAs [40,41,42]. The 5′-proximal ORF0 encodes a 28.9 kDa RNA silencing suppressor protein (P0), which is considered to function as an avirulence (AVr) determinant [40,43,44,45]. ORF1 encodes a 70.1 kDa protein (P1) expressed from genomic RNA [46] and ORF1-2 encodes the 118.7 kDa viral replication-associated protein P1-P2, including the RNA-dependent RNA polymerase (RdRp), which is expressed through a ribosomal frameshift [42,46]. The 3′-end comprises structural genes associated with viral encapsidation (ORF3), long-distance movement (ORF3a), cell-to-cell movement (ORF4), and aphid transmission (ORF5), all of which are expressed as subgenomic RNAs [46]. The viral ORF3 encodes the 22.4 kDa coat protein (CP; P3), while ORF4, which is nested within ORF3, encodes the 19.4 kDa movement protein (MP; P4) and is expressed as a frameshift. The ORF3a that encodes the P3a protein (5.2 kDa) is expressed through leaky scanning of the subgenomic RNA. The ORF5 is expressed through in-frame suppression of the ORF3 stop codon to yield a 77.2 kDa P3-P5 read-through domain (RTD). The CP-RTD is required for aphid transmission and in planta viral accumulation [38,47,48]. Finally, the 5′-terminal end is covalently bound to a genome-linked viral protein (VPg), but no poly(A)-tail or tRNA-like structures have been associated with the viral 3′-terminus [49,50].

The rapid genomic evolution observed among RNA plant viruses has been attributable to high rates of mutation, short generation times, and large population sizes [51,52]. The RdRp protein utilized for virus replication lacks proof-reading activity that can potentially result in a high mutation rate [53] and lead to recombination between viral genomes [54]. These evolutionary features of RNA viruses have been associated with enhanced virulence, differences in infectivity, transmission rate, symptom severity, and host range, including host-shifts [55,56].

The P0 silencing suppressor, encoded by the CLRDV ORF0, is the most variable coding region at both nucleotide and amino acid levels [32,40,57,58,59]. Despite the recent increased spread and emergence of CLRD disease and its detrimental effects on cotton productivity worldwide, there is little understanding of the genetic structure of CLRDV populations. Increased knowledge of genomic diversity and population structure of extant CLRDV isolates associated with cotton and non-cultivated host plants would help inform the selection of resistant cotton varieties while also improving the reliability of molecular detection and identification of variants required to elucidate the epidemiology locally and globally.

The objective of this study was to characterize the full-length genome of CLRDV isolates associated with cotton plants exhibiting disease symptoms observed in three US states affected by the 2017–2019 CLRDV outbreak. Here, eight new CLRDV genome sequences were determined by Sanger DNA sequencing and/or by high throughput sequencing (HTS) using Illumina technology. Analyses of the phylogenetic relationship, extent of recombination, and population structure were carried out for the 8 genomes and all available CLRDV sequences in the GenBank database. The goal was to inform local (US) and global perspectives of the dynamics surrounding the emergence of CLRDV and epidemiological implications.

## 2. Materials and Methods

### 2.1. The Plant Samples

Leaf samples were collected from cotton plants exhibiting mild to severe virus-like symptoms in Alabama (AL), Florida (FL), and Texas (TX) during 2019. The symptoms observed in some of the plants were similar to (while others were distinct from) those observed in CLRDV-infected cotton plants from AL during 2016–2017, an isolate referred to as CLRDV-AL [19,32]. The leaves were frozen immediately in liquid nitrogen and shipped by courier on dry ice (USDA-APHIS permit issued to the JK Brown Lab), to The University of Arizona, and stored at −80 °C.

### 2.2. Total RNA Isolation and RT-PCR Diagnostics

Total RNA was isolated from 100 mg of cotton leaves and petioles using a silica protocol adapted from [60] or with the Spectrum™ Plant Total RNA Kit (Sigma-Aldrich, St. Louis, MO, USA). The RNA was used as a template for reverse transcription polymerase chain reaction (RT-PCR) with primers designed to target the CLRDV ORF0/P0 or ORF4/P4 genomic regions [14,32]. The expected size amplicons were cloned into the pGEM-T easy plasmid vector (Promega Corp. Madison, WI, USA) used to transform competent cells of *Escherichia coli* DH5α and sent for Sanger DNA sequencing at Eton Biosciences (San Diego, CA, USA).

### 2.3. Determination of CLRDV Genomes

To determine the complete CLRDV genome sequences associated with mild to severe symptoms, total RNA was purified from eight representative cotton samples (Table 1), and genomic DNA was removed by treatment with RNase-free DNase I (Invitrogen, Carlsbad, CA, USA) according to the manufacturer’s instructions. The CLRDV genome sequence was obtained from four of the eight samples using high throughput sequencing (HTS) while the remaining four were determined by Sanger sequencing. For HTS, the cDNA library was constructed from total RNA following ribosomal RNA depletion and samples were subjected to Illumina RNAseq sequencing at Novogene Co. (Beijing, China). Removal of adapter sequences was carried out at Novogene Co., and low-quality bases were removed based on a sliding window size of 4 and Q score < 20. The de novo assembly of quality-trimmed raw Illumina reads was carried out using SeqMan NGen v.12 software (DNASTAR Inc. Madison, WI, USA) with a kmer parameter of 21, and maximum of 2 mismatches. To assess the assembled contig sequence quality, trimmed reads were mapped against the apparently full-length genomes of CLRDV using the Bowtie2 software [61] implemented in Geneious v.8.1.9 (Biomatters, San Diego, CA, USA). The assembled contigs were subjected to a BLASTn [62] search of the GenBank nucleotide database to establish preliminary virus identification. For Sanger sequencing, cDNA was synthesized with random hexamers using the PrimeScript 1st strand cDNA Synthesis Kit (Takara Bio USA Inc., Mountain View, CA, USA). A 2-μL cDNA aliquot was used in 25 μL PCR reaction with PrimeSTAR GXL DNA Polymerase (Takara Bio), as per manufacturer’s recommendation, to obtain the near complete CLRDV genome as a single DNA fragment with a primer pair designed based on aligned sequences of available CLRDV genomes in GenBank (Table S1). The obtained ~5.8 Kb sample-specific DNA bands were gel eluted with the Zymoclean Gel DNA Recovery Kit (Zymo Research Corporation, Irvine, CA, USA) and blunt-end cloned individually into the pJET1.2 plasmid vector (ThermoFisher Scientific, Waltham, MA, USA). Plasmid DNA was obtained from two positive recombinant clones per insert using the GenElute Five-Minute Plasmid Miniprep Kit (Sigma-Aldrich, St. Louis, MO, USA), as per manufacturer’s instructions, and these were Sanger sequenced and genome-walked until completion with additional genome-walking primers listed in Table S1. The overlapping genome sequence fragments were assembled with BioEdit v.7.2.5 program [63].

### 2.4. Sequencing of CLRDV 5′- and 3′-Untranslated Ends

To recover the 5′-untranslated region (UTR) of representative CLRDV isolates, cDNA synthesis was carried out for total RNA preparations using the reverse primer GSP1-CLRDV-5UTR-Rev (Table S1), according to the manufacturer’s protocol for the SuperScript IV First-Strand Synthesis System (Invitrogen, Carlsbad, CA, USA). Aliquots of cDNA were used as template for PCR amplification of CLRDV 5′-UTR with the primer pair CLRDV-P20-For/GSP2-CLRDV-5UTR-Rev (Table S1). Additionally, to recover the CLRDV 3′-UTR, total RNA was poly(A) tailed using the Poly(A) Tailing kit (Invitrogen, Carlsbad, CA, USA), and cDNA synthesis was carried out using oligo(dT) primer according to the manufacturer’s instructions accompanying the 3’ RACE kit (Invitrogen, Carlsbad, CA, USA). The cDNA was used as a template for PCR amplification of CLRDV 3′-UTR with primers GSP2-CLRDV-3UTR-For/anchor (3’ RACE kit) (Table S1).

The PCR amplification reactions were carried out using LongAmp Hot Start *Taq* 2X Master Mix (New England Biolabs, Ipswich, MA, USA), in a final volume of 25 μL: 12.5 μL of 2X LongAmp master mix, 0.2 μM of each primer, 1 μL of cDNA (template), and 10.5 μL of nuclease-free water. Cycling parameters were: initial denaturation for 2 min at 94 °C, followed by 35 cycles of denaturation at 94 °C for 20 s, annealing at 55 °C for 60 s, and extension at 65 °C for 30 s, and a final extension at 65 °C for 10 min. The amplicons were gel-purified and ligated to pGEM-T easy plasmid vector (Promega Corp. Madison, WI, USA) and transformed into competent cells of *E. coli* DH5α. Clones harboring inserts of the expected size were confirmed by colony PCR using M13 universal primers, and two or more clones per sample were sequenced bidirectionally (Eton Biosciences, San Diego, CA, USA).

### 2.5. Sequence Comparisons

Pairwise nucleotide sequence comparisons of the CLRDV complete genomes determined in this study and CLRDV sequences available in the GenBank database (Table S2), and CLRDV ORFs 0–5 were carried out using the Sequence Demarcation Tool (SDT) v.1.2 [64]. The pairwise amino acid (aa) similarity for the P0–P5 predicted protein sequences was calculated using the SDT software [64]. The species identification criterion of >10% divergence at the aa level for any poleroviral protein has been established for classification into the genus *Polerovirus* [36]. However, for CLRDV variants specifically, it has been proposed that isolates sharing <90% amino acid identity for ORF0, and >90% amino acid identity across the remaining proteins, represent different genotypes or variants of this species [32,58,59].

### 2.6. Phylogenetic Analysis

The CLRDV complete genome and ORF0 nucleotide (nt) sequences were aligned with the CLRDV sequences available in the GenBank database using MUSCLE [65]. The Bayesian phylogenetic trees were reconstructed using MrBayes v.3.2 [66] through the CIPRES web portal [67], and the GTR+I+G evolutionary model-of-best-fit. The analyses were carried out using two replicates of four chains each, and 10 million generations with sampling every 1000 generations. The first 2500 trees were discarded (burn-in), and the posterior probabilities [68] were determined based on a majority-rule consensus tree for the 15,000 remaining trees. Trees were edited in FigTree v.1.4 (tree.bio.ed.ac.uk/software/figtree) and Inkscape (https://inkscape.org/pt/). Furthermore, subpopulation structuring was tested by calculating Wright’s *F* fixation index (*Fst*) [69] using DnaSP v.6.10 [70]. The phylogenetic trees were midpoint-rooted because it was not possible to identify a polerovirus outgroup species that did not significantly confound the alignment and lead to a poorly resolved phylogeny.

### 2.7. Recombination Analysis

The eight newly determined CLRDV genomes were aligned with isolates previously reported in the US, and CLRDV-typical and -atypical sequences from South America. Non-treelike evolution analysis was carried out using the Neighbor-Net method implemented in SplitsTree v.4.10 [71]. The putative parental sequences and recombination breakpoints were predicted using the methods RDP, Geneconv, Bootscan, MAXCHI, Chimaera, SISCAN and 3Seq implemented in the RDP v.4.0 program [72]. The default settings were implemented for each different method, except that the sequences were considered linear instead of circular. The statistical significance threshold was set as a *P*-value lower than a Bonferroni-corrected cut-off of 0.05. Recombination events detected by at least five of the seven algorithms available in the RPD software with robust statistical support were accepted as reliable predictions.

### 2.8. Genetic Variability and Selection

The mean pairwise number of nucleotide differences per site (nucleotide diversity, π) was estimated using a 100-nt sliding window with a step size of 10 nt across the CLRDV genome subpopulation sequences using DnaSP v.6.10 [70]. Statistically significant differences amongst π values for each dataset/subpopulation were calculated by estimating the 95% bootstrap confidence intervals from 1000 nonparametric simulations using the Simpleboot package implemented in R (R Core Team) [73]. Additionally, the per-site nucleotide diversity was calculated for ORFs 0–5 sequences determined for each CLRDV subpopulation. The fixation index or *F_ST_* and *N_ST_* test statistics were calculated using DnaSP v.6.10 to compare the extent of genetic differentiation between populations/subpopulations and to characterize the biogeographical population structure, with potential *F_ST_* and *N_ST_* values ranging from 0 (no genetic differentiation) to 1 (complete differentiation) [69].

The aa sites evolving under positive or negative selection in ORFs 0–5 were predicted using the Fixed-Effect Likelihood (FEL) algorithm [74] implemented in DataMonkey (www.datamonkey.org; accessed on 15 July 2021). To reduce the likelihood of spurious selection estimates caused by recombination, subpopulation datasets were constructed for each recombination pattern predicted by both RDP4 and SplitsTree4, consisting of only non-recombinant sequences. The FEL *P*-values of <0.1 were considered significant. The mean ratio of non-synonymous to synonymous substitutions (*dN/dS*) was calculated using the Single-Likelihood Ancestor Counting (SLAC) method [74], where *dN/dS* = 1 predicts neutral evolution (no selection), and *dN/dS* > 1 or *dN/dS* < 1 indicates positive or negative selection, respectively [75].

## 3. Results

### 3.1. CLRDV Complete Genome Sequences

The eight apparently full-length newly determined CLRDV genome sequences were de novo and guided-assembled or Sanger-sequenced from representative samples displaying mild to severe symptoms. The Illumina sequences of 5865 to 5867 bp in length were derived from 13,043 to 147,639 reads from four isolates, having a depth of coverage of 336 to 3780 times (Table 1). The Sanger-derived sequences from the other four isolates were each 5579-nt in length, putatively missing 27-nt 5′UTR and 79-nt 3′-UTR sequences. The 5′- and 3′-UTRs of isolates CLRDV-AL [32], CLRDV-USA-AL-MC2, CLRDV-USA-FL-SC4, and CLRDV-USA-TX-CT3 were amplified by RT-PCR amplification (RACE), cloned, and Sanger sequenced, revealing fragments of 480 and 335 bp in length, respectively. The Sanger and Illumina-derived 5′- and 3′-UTRs shared >99.0% nt identity, indicating the two approaches used for sequencing and assembling the genome sequence data produced nearly identical and/or highly comparable genome sequences.

### 3.2. Pairwise Sequence Comparisons

The novel CLRDV complete genomes (*n* = 8) shared 96.5–100.0% nt identity with one another, and 94.5–8.1% nt identity with the next most closely related isolates for which sequences were available in GenBank, i.e., the CLRDV-AL variants previously reported in the US. The ORF0 has been recognized as the most variable coding region among the CLRDV isolates, with the new isolates sharing 93.3–100.0% nt identity among each other, and 89.4–99.7% identity with isolates from GenBank. Compared to the CLRDV-typical and -atypical genotypes reported from South America, the ORF0 nt sequences of the US isolates were 89.4–92.1% identical. As predicted, the P0 aa sequences exhibited the highest molecular variability among all of the viral coding regions, with the US CLRDV isolates sharing 90.4–100.0% aa similarity among each other, and 81.2–89.3% aa similarity with South American isolates. The three isolates collected in TX [CLRDV-USA-TX-CT3, CLRDV-USA-TXa, and CLRDV-USA-TXb (GenBank Accession Nos. OK185942-OK185944) shared the highest aa similarity with CLRDV isolates previously identified in cotton samples from TX (Accession MN872302), at 95.0–96.2%, whereas five isolates from TX, FL, and AL [CLRDV-USA-TXc, CLRDV-USA-TXd, CLRDV-USA-TX-CT2, CLRDV-USA-AL-MC2, and CLRDV-USA-FL-SC4 (GenBank Accession Nos. OK185945-OK185946, OK185941, OK185939, and OK185940)] were more closely related to the CLRDV-AL genotype previously reported from AL and Georgia (Accession Nos. MN071395 and MT800932), at 95.0–99.6% aa similarity. For ORFs 2–5, the nt and aa sequence pairwise distance estimates exceeded 92.0% and 90.0% identity, respectively, except for isolate CLRDV-GA40 from Georgia (GA), which showed aa similarities between 87.5–91.5 and 87.8–88.5% for ORF2, compared to CLDRV-US and CLRDV-SA isolates, respectively (Table 2). Thus, the newly characterized genomes most closely resembled the first CLRDV genome sequences discovered in the US, the CLRDV-AL genotypes [18,32].

### 3.3. Phylogenetic Relationship

**Figure 1:**
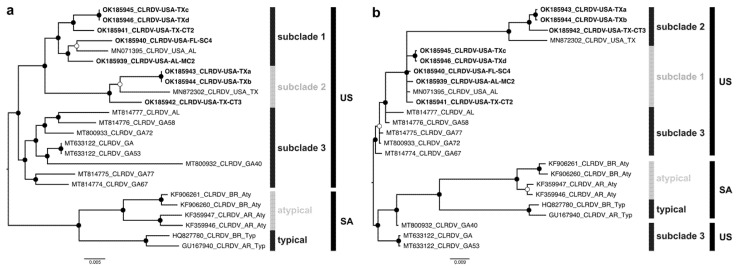
Midpoint-rooted Bayesian phylogenetic trees reconstructed from complete genomes (**a**) and ORF0 nucleotide sequences (**b**) of cotton leafroll dwarf virus (CLRDV). Posterior probability values are represented by filled (0.95–1.00) and empty (0.80–0.94) circles near to the branch nodes. Sequences determined in this study are highlighted in bold font.3.4. Recombination.

Bayesian phylogenetic trees were reconstructed from the apparently full-length genome and ORF0 nt sequences of the new CLRDV isolates (*n* = 8) and sequences previously reported in the US (*n* = 9) and South America (*n* = 6). The CLRDV-US isolates formed a monophyletic group consisting of three sister clades. The subclade 1-US harbored sequences from TX, AL, and FL (*n* = 6), subclade 2-US isolates found only in TX (*n* = 4), while the subclade 3-US was comprised of sequences in GA and AL (*n* = 7) (Figure 1a). Furthermore, the CLRDV sequences from the US (CLRDV-AL) and South America (CLRDV-typical and -atypical) formed distinct phylogenetic groups, reinforcing the hypothesis that extant CLRDV-US genotypes or subpopulations are divergent and geographically structured in relation to other isolates recognized so far. The CLRDV-typical and -atypical variants grouped into two subclades (Figure 1a). The CLRDV ORF0 and complete genome trees were incongruent, with some isolates from GA being more closely related to CLRDV reported in SA (Figure 1b).

**Figure 2:**
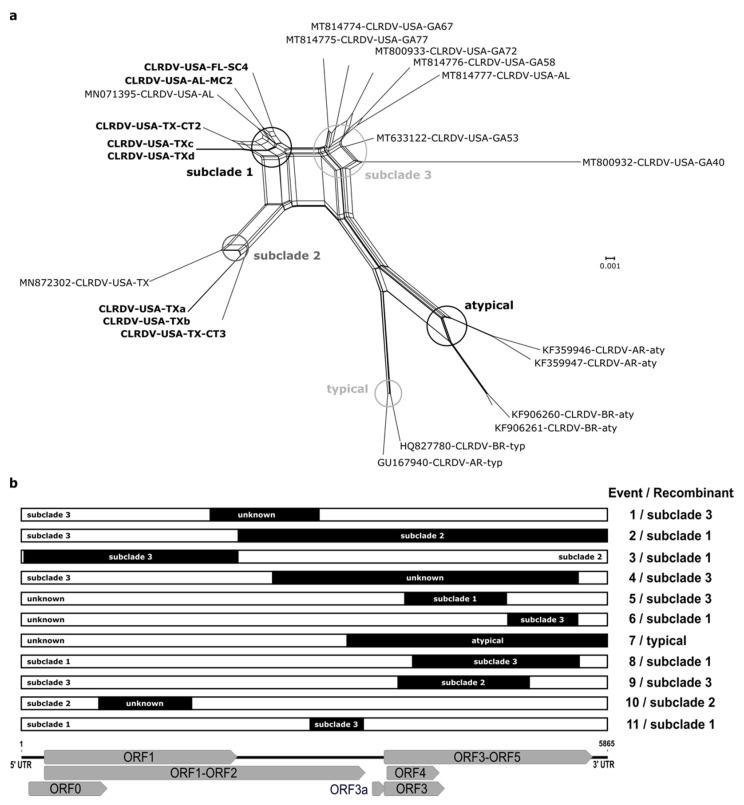
Reticulate phylogenetic network constructed using the Neighbor-Net method (**a**) and recombination events detected in RDP4 (**b**) based on analysis of aligned complete genomes of cotton leafroll dwarf virus (CLRDV). (**a**) Reticulation among the viral isolates is shown by parallel paths instead of a bifurcating evolutionary tree indicative of putative recombination. The branch internodes for the phylogenetic subclades are circled. (**b**) The regions highlighted in black correspond to the portion donated by the predicted minor parent, while the remaining portions represent the major parent of each independent recombination event. The CLRDV genome organization is shown in relation to the aligned sequences.

Reticulate (non-treelike) branching patterns were detected for the CLRDV complete genomes using the Neighbor-Net distance-based method. Several parallel/boxes paths were observed in the network, indicating conflicting signals probably caused by recombination events. The reticulation was pronounced for the different subclades from the US (subclades 1–3) and South America (subclades typical and atypical), suggesting that the evolution of these CLRDV sequences may have been shaped by genetic recombination. Additionally, the long branches associated with the CLRDV isolates indicated that these genomes harbored more mutations than isolates grouping in clades having shorter branch lengths (Figure 2a).

To predict possible recombination among CLRDV isolates, and identify the respective breakpoints and parents, all available CLRDV complete genomes were aligned and analyzed using the RDP software package. At least 11 independent recombination events were predicted among the CLRDV isolates, with most recombination breakpoints having occurred in the second half of ORF2 and ORF5, and in the first half of ORF3 (events 1–9 and 11) (Figure 2b; Table S3). The CLRDV-GA isolates (subclade 3-US) were identified as putative recombinants in three events (1, 4, and 9) and as possible parental sequences in eight recombination events (1–4, 6, 8, 9, and 11). Further, CLRDV isolates in subclade 1-US were identified as recombinants, with sequences of isolates from TX (subclade 2-US) or GA (subclade 3-US) implicated as possible minor and major parents (events 2, 3, 6, 8, and 11). The CLRDV-typical isolates from Brazil (BR) and Argentina (AR) were also identified as putative recombinants, with CLRDV-atypical (BR) as the predicted minor parent, but the major parent was unidentified (Figure 2b; Table S3).

### 3.4. Population Genetics

The mean pairwise number of nucleotide differences per site (nucleotide diversity, π) was estimated for the CLRDV complete genomes using DnaSP v.6. The CLRDV sequences were grouped as seven subpopulations, representing isolates extant in South America (SA; *n* = 6), typical-SA (*n* = 2), atypical-SA (*n* = 4) and the US (*n* = 17), subclade 1-US (*n* = 6), subclade 2-US (*n* = 4), and subclade 3-US (*n* = 7). Based on *Fst* and *Nst* values, extensive population structure was evident between most of the subpopulations, indicative of genetic divergence. In contrast, relatively low *Fst* and *Nst* indices were obtained when subclades 1-US and 3-US were compared with the entire US subpopulation, at 0.07 and 0.06, respectively (Table 3). Finally, a comparison of atypical-SA with SA subpopulations revealed negative *Fst* and *Nst* values of − 0.07 (Table 3).

**Figure 3:**
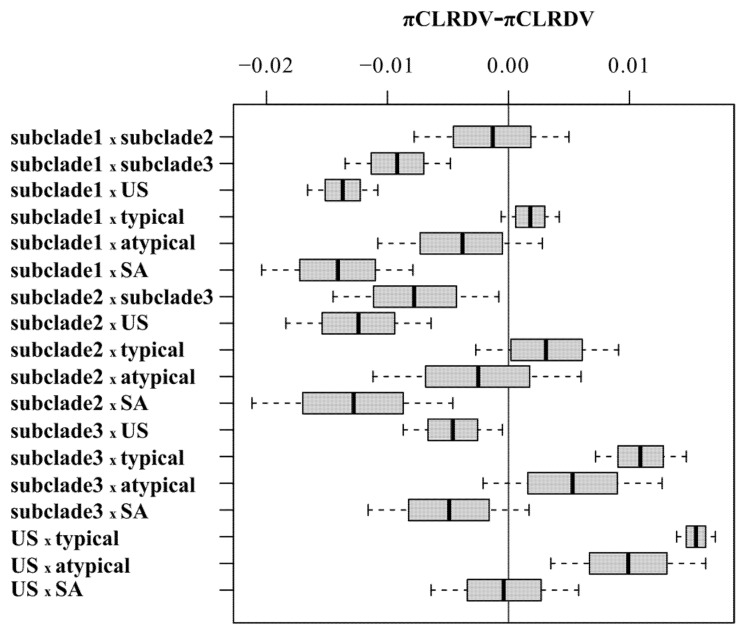
The statistical significance of the differences amongst the mean pairwise number of nucleotide differences per site (π), as evaluated by 95% bootstrap confidence intervals (CIs) calculated using 1000 nonparametric simulations. No statistically significant differences observed for CI values including zero (grey line) for pairwise comparisons based on the complete genome of cotton leafroll dwarf virus (CLRDV) subpopulations.

**Figure 4:**
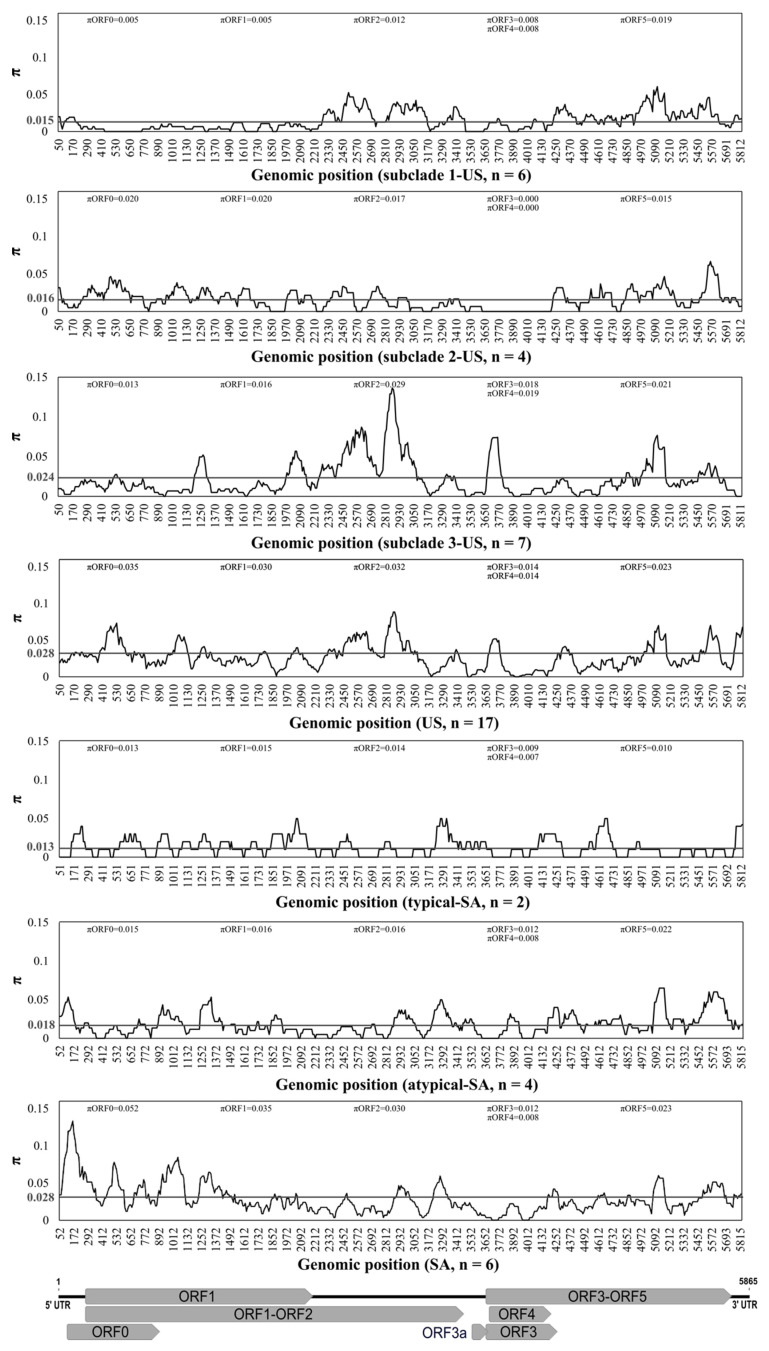
Nucleotide diversity across the complete genomes of cotton leafroll dwarf virus (CLRDV) isolates, calculated using DnaSP6 on a 100-nucleotide sliding window with a step size of 10 nucleotides. The horizontal lines (in grey) correspond to the genome-wide average nucleotide diversity for the full-length sequences within each subpopulation. The CLRDV genome organization is shown in relation to the alignment.

Statistically significant differences for pairwise comparisons of π values calculated from full-length genomes of CLRDV subpopulations of different sample sizes were analyzed with a 95% bootstrap confidence interval (CIs) using the approach described by [73], where no statistically significant differences were observed for CI values including zero (grey line) (Figure 3). The nucleotide diversity analysis of the complete CLRDV genomes revealed that genetic variability was unevenly distributed across the viral genomes (Figure 4). The lowest nucleotide diversity for isolates affiliated with the subclades 1-US and 3-US resided within the 5′- portion of the genome, which encodes ORF0, whereas ORF0 was the most variable coding region among all of the CLRDV-SA (*n* = 6) and CLRDV-US (*n* = 17) populations (Figure 4). The second half of ORFs 2 and 5, and the first half of ORF3 harbored the greatest nucleotide diversity among subclade 3-US isolates (Figure 4). Finally, the diversity was highest among US and SA populations, compared to individual subpopulations, an observation indicative of interpopulation variation. Although CLRDV genome sequences from South America were under-represented among the total sequences available, the genomic variability among the South American (*n* = 6) and US (*n* = 17) CLRDV isolates was comparable (π*_SA_* = 0.02867 and π*_US_* = 0.02831; Figure 3 and Figure 4).

The potential recombination events among different CLRDV genomes were predicted using RDP4 and SplitsTree4 (Figure 2), and selective forces acting on CLRDV evolution were estimated based on subpopulation datasets consisting of only nonrecombinant sequences. Using the SLAC method, the *dN/dS* mean ratios for ORFs 0–3 and 5 were < 1 for nearly all datasets, which is indicative of purifying selection. The exception was ORF0 of the atypical-SA, with a *dN/dS* ratio of 1.63, indicative of positive selection. In contrast, the *dN/dS* values for ORF4 among all of the CLRDV isolates range from 0.98–1.26, revealing widespread positive selection on this viral ORF (Table 4). Further, most statistically significant aa sites identified in ORFs 0–5 were shown to be evolving under purifying selection. Only a small number of statistically significant aa sites exhibited positive selection, and these were distributed throughout ORFs 2–5 (Table 4). These results indicated that negative selection was the most important selective force acting on viral coding regions.

## 4. Discussion

Cotton is one of the most economically important crops worldwide, and significant yield loses caused by cotton leafroll dwarf virus (the causal agent of cotton blue- and cotton blue-like diseases) have been reported in Brazil and Argentina [9,10,12,58,59], East Timor [13,14], India [15], Thailand [16], and, most recently, in the US [19,32]. Since 2016–2017, CLRDV has emerged and spread quickly from those sites in cotton-growing states in the southern and south-central US [19,20,21,22,23,24,25,26,27,28,29,30,32]. Despite the potential for economic importance of CLRDV, little is known about CLRDV genome-level variability and the forces that may be acting upon it. In this study, the high nucleotide diversity documented among CLRDV isolates was primarily influenced by mutation and recombination, leading to geographically structured viral populations, suggestive of admixture through the introduction of multiple variants and/or multiple introductions in or near the same foci, potentially followed by short and/or long-distance spread by the aphid vector.

Although cotton plants exhibiting CBD-like symptoms have been recognized in several cotton-growing areas of the world since the 1930′s [7,8,9,10], the etiological agent was identified in 2005 when partial sequences of the polerovirus CLRDV were associated with symptomatic cotton in Brazil [12]. The first CLRDV complete genome sequence was reported five years later [17]. Most recently, several genomes of a new CLRDV variant, CLRDV-AL, were reported in the US [21,25,32,57]. In this study, eight CLRDV full-length genome sequences were determined by HTS and Sanger sequencing of isolates collected from commercially grown cotton plants in AL, FL, and TX. The pairwise nt and aa comparisons for the eight CLRDV genomes and the associated ORFs 0–5 indicated that all isolates from the US were most closely related to the CLRDV-AL genotype, and therefore only distantly related to known isolates from South America. Previously, the CLRDV isolates from the US were reported to cluster into two divergent subclades, with the TX isolate (Accession MN872302) representing the most divergent among all genome sequences available thus far from US isolates [57]. However, with the addition of eight newly determined genome sequences representing isolates from three different states, the evolutionary relationship among CLRDV-AL isolates revealed at least three subclades, herein, referred to as subclades 1–3-US. The latter isolates clustered as a monophyletic group distinct from the genome sequences from South America. Based on the high *F_ST_* (0.32–0.52) and *N_ST_* (0.32–0.51) estimates, CLRDV isolates exhibited phylogenetic structure at the subclade level.

Based on phylogenetic analysis of the ORF0, several CLRDV isolates from Georgia grouped more closely with CLRDV-SA than with the other US isolates. This observation could suggest that gene flow may have occurred between CLRDV isolates extant in the two geographically distant locales. However, the *F_ST_* (0.42) and *N_ST_* (0.42) indices, based on complete genome sequences, revealed a high degree of genetic differentiation, suggesting that gene flow between South America and the US has been insufficient to group these isolates as a unified population. The relatively low *F_ST_* and *N_ST_* values observed between the subclades 1-US (0.07) and 3-US (0.06), compared to the US population, may suggest that the variability frequencies in these subpopulations are similar, while the negative *F_ST_* and *N_ST_* (−0.07) between atypical-SA and SA could indicate that most variability derives from within subpopulation-level differences [76]. Even so, additional CLRDV complete genome sequences are needed to better resolve the population structure and predict the recent origin(s) of CLRDV in both the US and South America. To accomplish this, additional isolates from cotton and from diverse wild host species from different locations throughout the southern US cotton growing states are needed. Additionally, a larger, more representative CLRDV genome database would aid in predicting the recent origin(s) of US isolates and their route(s) of spread within the US and their relationships to CLRDV from Asia and South America.

Recombination provides a vital evolutionary mechanism shaping the molecular variability of plant viruses, including poleroviruses [77]. The intergenic regions (IR) between ORF2 and ORF3 of poleroviruses have been reported as a hotspot of recombination [18,78,79,80,81]. Preliminary analysis of partial CLRDV sequences suggested that the CLRDV-atypical isolates in South America emerged through recombination involving the 5′-block of the viral genome [18]. A complex recombination pattern among CLRDV-GA genomes was recently reported, where these isolates were detected as putative recombinant or parental sequences in seven of ten independent recombination events [57]. The results described here are consistent with previous findings, with at least 11 independent recombination events being predicted among CLRDV isolates, and CLRDV-GA involved as potential recombinant or parental genomes in eight of these events. Recombination breakpoints detected across the viral genomes were mainly localized between ORF2 and ORF3, and ORF5, underscoring the importance of recombination as an evolutionary mechanism underlying CLRDV diversification. In another study, CLRDV was detected in 23 weed species, representing 16 botanical families, naturally occurring in cotton fields in GA, US [34], pointing to the potential for a much greater-than-expected CLRDV host range, and widespread distribution of year-round virus reservoirs. Wild plant species are known to act as mixing vessels that can support high viral diversity, and mixed infections, facilitating virus diversification through recombination and emergence of new viral genotypes [82]. This may explain, in part, why most recombination events were observed among CLRDV in GA. Determining CLRDV genome sequences from wild reservoir hosts would aid in understanding their potential for driving evolution of recombinant genotypes.

Relatively low genome variability has been observed among CLRDV ORF2 and ORF3 [18]. In the context of cultivated cotton and breeding efforts to develop tolerance or resistance to CLRDV, it may not be surprising that CLRDV ORF0, which is known to function as a suppressor of plant host gene silencing, is the most variable coding region among cotton-infecting isolates of CLRDV [32,34,40,43,44,57,58,59]. Here, high per site nucleotide diversity was observed among the CLRDV populations from the US and South America, with ORF0 exhibiting the greatest extent of diversification. Interestingly, the CLRDV isolates associated with the phylogenetic subclade 3-US (π = 0.024) had significantly higher nucleotide diversity indices compared to sequences in subclades 1-US (π = 0.015) and 2-US (π = 0.016), for which ORF2 (π = 0.029) was the most divergent coding region. These observations might be explained by recombination potentially having occurred among subclade 3-US isolates, which were predicted as putative recombinants or parents in nine of 11 independent recombination events. Several recombinants were also previously identified among the CLRDV sequences from GA (subclade 3-US) [57], adding further support for the hypothesis that both mutation and recombination influenced CLRDV evolution.

Selective forces acting on viral coding regions are expected to vary, particularly for multifunctional proteins [83]. Although high variability was observed among the CLRDV isolates, most prominently in ORF0/P0, negative selection (*dN/dS* < 1.0) appeared to have expunged most nonsynonymous mutational effects in the CLRDV ORFs, owing to deleterious effects [83]. Consequently, most mutations detected in viral coding regions showed no additional aa sequence modifications, a scenario that is consistent with conserved protein structure and function. The CLRDV 5′ block of genes function in viral replication and in the suppression of host gene silencing, whereas the 3′ block encodes proteins involved in genome encapsidation, cell-to-cell movement, and aphid vector transmission [38,40,42,46,47,48]. Conservation of the viral proteins involved in replication and aphid transmission is of particular importance for virus survival and spread. Notably, the viral movement protein (ORF4) of phylogenetic subclade 1-US and atypical-SA, and ORF0 of atypical-SA were found to be under positive or diversifying selection (*dN/dS* > 1.0). In contrast, ORF5 showed the lowest *dN/dS* value, a result consistent with its role in aphid-mediated transmission. Although these results suggested that either multiple CLRDV variants were introduced into the US at one time or that distinct isolates were introduced at different times, followed by subsequent diversification, it is not yet possible to determine the extent to which cotton genotype may have differentially influenced viral diversification. Likewise, the timespan(s) between the initial introduction(s) to the present cannot be determined without additional, broad-scale sampling of the southern US cotton-growing states. Overall, the small sample size represented by the two predominant CLRDV genome types extant in the Americas (*n* = 6), together with the lack of complete CLRDV genome sequences for Asian or African isolates precludes a more extensive review of population dynamics. Similarly, understanding the relationship between genomic variability and diversification in the context of local and global epidemiological patterns of spread will require an analysis of CLRDV sequences determined from all known host species and locations where the virus is now known to occur.

## 5. Conclusions

The introduction of CLRDV into previously uninfected commercial cotton-growing regions, and/or the emergence of new viral genotypes in the US and elsewhere, present economic threats to the cotton industry, with yield losses reported to be as great as 80% in susceptible varieties [18]. Although CLRDV-resistant cotton varieties have been developed that provide protection against the original CLRDV-typical isolates, the so-named resistant-breaking variant, CLRDV-atypical, has emerged in Brazil [59] and Argentina [58]. Considering that SA and US isolates represent two divergent, geographically distinct subpopulations, and interactions between cotton varieties and CLRDV variants of the US subclades 1–3 are unstudied, the movement of CLRDV isolates between these infection foci should be avoided to mitigate their spread to locations where they do not occur naturally. This new knowledge of genetic variability in CLRDV populations is expected to inform cotton breeding efforts to further develop CLRDV-resistant germplasm. Challenging the most promising cotton genotypes with the different CLRDV variants will aid in characterizing genetic resistance in cotton. Finally, additional research is required to better elucidate CLRDV epidemiology, including routes or pathways of short- and long-distance spread, understand the relationship between genome variation and recombination among CLRDV isolates infecting different cotton genotypes and wild plant host species, as well as the role of cotton aphid transmission in CLRDV diversification. Even so, this new knowledge is expected to advance the development of reliable tools for molecular detection, support resistance breeding efforts, and provide initial epidemiological clues about the distribution of the two main CLRDV groups extant in the US or SA.

## Figures and Tables

**Table 1:** Cotton leafroll dwarf virus (CLRDV) isolates recovered from symptomatic cotton plants using high throughput sequencing or Sanger sequencing.

Isolate	Location	Symptoms	PCR-ORF0	# of Reads	Coverage	Reference
CLRDV-USA-AL-MC2 ^1^	Alabama	Severe	Positive	33,141	850	This study
CLRDV-USA-AL-SC4 ^1^	Alabama	Mild	Positive	16,964	435	This study
CLRDV-USA-TX-CT2	Texas	Severe	Positive	13,043	336	This study
CLRDV-USA-TX-CT3 ^1^	Texas	Severe	Positive	147,639	3,780	This study
CLRDV-USA-TXa	Texas	Severe	Positive	Sanger-derived		This study
CLRDV-USA-TXb	Texas	Severe	Positive	Sanger-derived		This study
CLRDV-USA-TXc	Texas	Severe	Positive	Sanger-derived		This study
CLRDV-USA-TXd	Texas	Severe	Positive	Sanger-derived		This study
CLRDV-USA-AL ^1^	Alabama	Severe	Positive	-	-	Avelar et al., 2020

^1^ Representative CLRDV isolates for which viral 5′ and 3′ UTRs were determined.

**Table 2:** Nucleotide and amino acid identities for the complete genome and open reading frame (ORF) sequences of cotton leafroll dwarf virus (CLRDV) isolates from the US and South America (SA).

Genomes—Nucleotide/Amino Acid			ORF0—Nucleotide/Amino Acid		
	CLRDV-SA	CLRDV-US		CLRDV-SA	CLRDV-US
CLRDV-SA	95.9–99.5/- ^1^		CLRDV-SA	91.1–99.5/87.0–98.1	
CLRDV-US	94.0–96.2/-	94.5–100.0/-	CLRDV-US	89.4–94.3/81.6–90.4	92.1–100.0/90.8–100.0
**ORF1—Nucleotide/Amino Acid**			**ORF1-ORF2—Nucleotide/Amino Acid**		
	CLRDV-SA	CLRDV-US		CLRDV-SA	CLRDV-US
CLRDV-SA	94.3–99.5/93.8–99.5		CLRDV-SA	95.5–99.4/93.3–99.1	
CLRDV-US	92.4–95.5/90.3–95.0	93.8–100.0/91.6–100.0	CLRDV-US	92.9–96.2/87.8–94.7	92.5–100.0/87.5–100.0
**ORF3—Nucleotide/Amino Acid**			**ORF3a—nucleotide/amino acid**		
	CLRDV-SA	CLRDV-US		CLRDV-SA	CLRDV-US
CLRDV-SA	97.9–100.0/98.5–100.0		CLRDV-SA	97.8–100.0/100.0	
CLRDV-US	95.0–98.3/95.5–100.0	96.2–100.0/95.5–100.0	CLRDV-US	97.1–100.0/100.0	97.8–100.0/100.0
**ORF4—Nucleotide/Amino Acid**			**ORF5—Nucleotide/Amino Acid**		
	CLRDV-SA	CLRDV-US		CLRDV-SA	CLRDV-US
CLRDV-SA	98.1–100.0/95.4–100.0		CLRDV-SA	96.7–99.7/96.8–100.0	
CLRDV-US	94.9–98.9/89.7–97.1	96.0–100.0/92.5–100.0	CLRDV-US	94.8–96.8/94.1–98.3	96.5–100.0/95.7–100.0

^1^ No amino acid sequence comparisons were carried out for CLRDV complete genomes.

**Table 3:** Genetic structure among cotton leafroll dwarf virus (CLRDV) subpopulations.

Subpopulations		*Nst* ^1^	*Fst* ^1^
subclade 1-US	subclade 2-US	0.52	0.51
subclade 1-US	Subclade 3-US	0.31	0.31
subclade 1-US	US	0.07	0.07
subclade 1-US	typical-SA	0.72	0.72
subclade 1-US	atypical-SA	0.67	0.67
subclade 1-US	SA	0.56	0.56
subclade 2-US	subclade 3-US	0.51	0.50
subclade 2-US	US	0.27	0.27
subclade 2-US	typical-SA	0.71	0.71
subclade 2-US	atypical-SA	0.66	0.66
subclade 2-US	SA	0.56	0.55
subclade 3-US	US	0.06	0.06
subclade 3-US	typical-SA	0.62	0.62
subclade 3-US	atypical-SA	0.56	0.55
subclade 3-US	SA	0.45	0.45
US	typical-SA	0.58	0.57
US	atypical-SA	0.53	0.52
US	SA	0.42	0.41
typical-SA	atypical-SA	0.60	0.60
typical-SA	SA	0.26	0.26
atypical-SA	SA	− 0.07	− 0.07

^1^ The *F_ST_* and *N_ST_* values range from 0 for no genetic differentiation, to 1 for complete differentiation between populations.

**Table 4:** Nonsynonymous to synonymous substitution rates (*dN/dS*) and number of positively and negatively selected sites in the ORFs 0–5 of cotton leafroll dwarf virus (CLRDV) subpopulations using Single-Likelihood Ancestor Counting (SLAC) and Fixed-Effect Likelihood (FEL), respectively.

Dataset	Gene	SLAC (*dN/dS*)	FEL (# of Sites)	
			Negative	Positive
CLRDV-subclade1-US	ORF0	0.52	2	0
	ORF1	0.36	3	0
	ORF2	0.15	33	0
	ORF3	0.16	3	0
	ORF4	1.19	1	0
	ORF5	0.19	30	0
CLRDV-subclade2-US	ORF0	0.36	8	0
	ORF1	0.28	27	0
	ORF2	0.19	54	1
	ORF3	− ^1^	−	−
	ORF4	−	−	−
	ORF5	0.24	20	0
CLRDV-subclade3-US	ORF0	0.67	3	0
	ORF1	0.55	17	0
	ORF2	0.47	54	3
	ORF3	0.50	6	0
	ORF4	0.98	1	1
	ORF5	0.38	29	0
CLRDV-atypical-SA ^2^	ORF0	1.63	1	0
	ORF1	0.33	13	0
	ORF2	0.25	32	0
	ORF3	0.16	5	1
	ORF4	1.26	1	0
	ORF5	0.15	16	1

^1^ Analysis was not performed because the dataset was comprised by one only haplotype. ^2^ The selection analysis was not carried out for the CLRDV-typical-SA isolates because at least three different haplotypes are required.

## Data Availability

The new viral complete genome sequences are available NCBI-GenBank (https://www.ncbi.nlm.nih.gov/genbank/) under accession numbers OK185939-OK185946.

## Supplementary Materials

The following are available online at https://www.mdpi.com/article/10.3390/v13112230/s1, Table S1: Primers used for the genome amplification of complete cotton leafroll dwarf virus (CLRDV) and its 5′/3′ untranslated regions (UTRs) by random amplification of cDNA ends; Table S2: Cotton leafroll dwarf virus (CLRDV) genome sequences retrieved from NCBI-GenBank; Table S3: Predicted recombination events detected for cotton leafroll dwarf virus (CLRDV) isolates.
