# Supplementary material for: Cotton Leafroll Dwarf Virus US Genomes Comprise Divergent Subpopulations and Harbor Extensive Variability
Source: Viruses. 2021 Nov 5;13(11):2230. doi: 10.3390/v13112230 (PMC8618375; doi:10.3390/v13112230)
Supplement: Supplementary file 1 [file viruses-13-02230-s001.zip › viruses-1418952 - supplementary-done.pdf]

viruses MDPI

Supplementary Material

Table S1. Primers used for amplification of the full-length Cotton leafroll dwarf virus (CLRDV) genome and of the 5′/3′ untranslated regions (UTRs) by random amplification of the ends of viral cDNAs.

Primer[1]	Primer sequence (5′–3′)[2]	Target	Amplicon	Reference
Genome				
CLRDV-v28	ACACTTGAGACATAACTCGCTT	5′ UTR	5759 bp	This Study
CLRDV-c5786	CTTYCRGATTCGTTCCTGTT	3′ UTR		
Genome-walking				
CLRDV-v803	GCTGCTAYACCAAAGCCAAATC	ORF0	5104 bp	This Study
CLRDV-c5104	CTGCCGATTTGGAAGAGGAA	ORF3-ORF5		
CLRDV-v1617	CTCTGCWCCACCACTTTCC	ORF1	2724 bp	This Study
CLRDV-c4340	CATTGCGGATTTCCCTCATAAC	ORF3-ORF5		
CLRDV-v2438	GCTCTTGCCAGTCCTTTCT	ORF1-ORF2	1079 bp	This Study
CLRDV-c3516	CCAGTGTTAGGCTGTGATCTT	ORF3a		
5′ UTR				
GSP1-CLRDV-5UTR-Rev	CCGCGAGTGCAGAGATACTC	ORF0	520 bp	Distéfano et al., 2010
GSP2-CLRDV-5UTR-Rev	CGACCAGAGAGCGAGTAGCA		480 bp	Distéfano et al., 2010
3′ UTR				
GSP1-CLRDV-3UTR-For	AACCAACACGATCAATTTCA	ORF5	510 bp	Distéfano et al., 2010
GSP2-CLRDV-3UTR-For	AGAAGCCGCTCCAGTTGGGG		335 bp	Distéfano et al., 2010
CLRDV 5′ UTR				
CLRDV-P20-For	ACAAAAGAACGATAGAGGGGTTGT	5′ UTR	-	Avelar et al., 2019

[1]Virion (v)- or complimentary (c)-sense position of each primer is based on isolate CLRDV-AL-USA (MN071395). GSP, gene specific primer; [2]T R = A/G; Y = C/T; W = A/T.

Table S2. Cotton leafroll dwarf virus genome sequences downloaded from the NCBI-GenBank database.

Species	Isolate	GenBank Accession #	Host	Country
Cotton leafroll dwarf virus	USA-AL	MN071395	Cotton	USA
Cotton leafroll dwarf virus	USA-AL	MT814777	Cotton	USA
Cotton leafroll dwarf virus	USA-TX	MN872302	Cotton	USA
Cotton leafroll dwarf virus	USA-GA40	MT800932	Cotton	USA
Cotton leafroll dwarf virus	USA-GA53	MT633122	Cotton	USA
Cotton leafroll dwarf virus	USA-GA58	MT814776	Cotton	USA
Cotton leafroll dwarf virus	USA-GA67	MT814774	Cotton	USA
Cotton leafroll dwarf virus	USA-GA72	MT800933	Cotton	USA
Cotton leafroll dwarf virus	USA-GA77	MT814775	Cotton	USA
Cotton leafroll dwarf virus	AR-typical	GU167940	Cotton	Argentina
Cotton leafroll dwarf virus	BR-typical	HQ827780	Cotton	Brazil
Cotton leafroll dwarf virus	BR-atypical	KF906260	Cotton	Brazil
Cotton leafroll dwarf virus	BR-atypical	KF906261	Cotton	Brazil
Cotton leafroll dwarf virus	AR-atypical	KF359946	Cotton	Argentina
Cotton leafroll dwarf virus	AR-atypical	KF359947	Cotton	Argentina

Table S3. Predicted recombination events detected for cotton leafroll dwarf virus (CLRDV) isolates.

Event	Breakpoints[1] Begin	Breakpoints[1] End	Recombinant	Parents Minor	Parents Major	Methods[4]	*P* value[5] Highest	*P* value[5] Lowest
1	1956[3] **ORF1**	3035 **ORF2**	[2]MT800932_CLRDV_GA40	Unknown	MT814777_CLRDV_AL	RGBMC**S***3S*	6.01×10^{-14}	3.76×10^{-40}
2	2208[3] **ORF2**	5866[3] **3UTR**	[2]OK185941_CLRDV_USA_TX_CT2<br>OK185945_CLRDV_USA_TXc<br>OK185946_CLRDV_USA_TXd	OK185942_CLRDV_USA_TX_CT3	MT814776_CLRDV_GA58	R**G**BMCS*3S*	9.40×10^{-3}	1.03×10^{-20}
3	38[3] **5UTR**	2210 **ORF2**	[2]OK185939_CLRDV_USA_AL_MC2<br>OK185940_CLRDV_USA_FL_SC4<br>MN071395_CLRDV_USA_AL	MT814776_CLRDV_GA58	OK185942_CLRDV_USA_TX_CT3	R**G**BMCS*3S*	2.17×10^{-10}	5.09×10^{-20}
4	2502 **ORF2**	5578[3] **ORF5**	MT814775_CLRDV_GA77<br>MT814774_CLRDV_GA67	Unknown	MT814777_CLRDV_AL	**R**G*M*C3S	1.07×10^{-4}	9.26×10^{-9}
5	3800[3] **ORF3**	4935 **ORF5**	[2]MT814777_CLRDV_AL	OK185941_CLRDV_USA_TX_CT2	Unknown	RG*B*M**C**S3S	3.15×10^{-4}	7.47×10^{-8}
6	4957[3] **ORF5**	5539 **ORF5**	[2]MN071395_CLRDV_USA_AL	MT633122_CLRDV_GA53	Unknown	R**G**BMCS*3S*	2.90×10^{-2}	1.74×10^{-8}
7	3279 **ORF2**	5865[3] **3UTR**	[2]GU167940_CLRDV_AR_Typ<br>HQ827780_CLRDV_BR_Typ	KF906260_CLRDV_BR_Aty	Unknown	**R**B*M*CS3S	1.00×10^{-4}	7.41×10^{-9}
8	3896 **ORF3**	5546[3] **ORF5**	OK185940_CLRDV_USA_FL_SC4	MT814776_CLRDV_GA58	OK185939_CLRDV_USA_AL_MC2	B*M*C**S**3S	4.29×10^{-2}	5.18×10^{-8}
9	3704 **ORF3**	5128 **ORF5**	MT800932_CLRDV_GA40	MN872302_CLRDV_USA_TX	MT814776_CLRDV_GA58	R**G**BMC*3S*	4.17×10^{-3}	4.46×10^{-7}
10	808 **ORF0**	1762 **ORF1**	[2]OK185942_CLRDV_USA_TX_CT3	Unknown	MN872302_CLRDV_USA_TX	*R***G**BMCS3S	4.53×10^{-2}	2.63×10^{-5}
11	2888 **ORF2**	3440 **ORF2**	[2]MN071395_CLRDV_USA_AL<br>OK185940_CLRDV_USA_FL_SC4<br>OK185939_CLRDV_USA_AL_MC2	MT814774_CLRDV_GA67	OK185941_CLRDV_USA_TX_CT2	R**M**C*S*3S	1.08×10^{-2}	4.00×10^{-4}

[1]Numbering starts at the 5′ end of the minus-strand primer-binding site and increases clockwise; [2]The recombinant sequence may have been misidentified (one of the identified parents might be the recombinant); [3]Breakpoint could not be precisely pinpointed; [4]R, RDP; G, GeneConv; B, Bootscan; M, MaxChi; C, Chimera; S, Siscan; 3S, 3SEQ; [5]The reported *P*

values are for the methods indicated in bold and italicized, and they are the lowest and highest *P* values calculated for the region in question, respectively.
